# Persistance des épidémies de konzo à Kahemba, République Démocratique du Congo: aspects phénoménologiques et socio-économiques

**DOI:** 10.11604/pamj.2014.18.213.4572

**Published:** 2014-07-15

**Authors:** Daniel Okitundu Luwa E-Andjafono, Guy Bumoko Makila-Mabe, Marie-Thérèse Sombo Safi Ayanne, Jackin Kambale Kikandau, Nicole Mashukano, Théodore Kazadi Kayembe, Dieudonné Mumba Ngoyi, Michael Joseph Boivin, Jean-Jacques Tamfum-Muyembe, Jean-Pierre Banea Mayambu, Désiré Tshala-Katumbay

**Affiliations:** 1 Centre Neuro-psycho-pathologique, Département de Neurologie, RDC; 2 Sciences de Base, Université de Kinshasa (UNIKIN), RDC; 3 Programme National de Nutrition, RDC; 4 Département de Médecine Tropicale, UNIKIN & Institut National de Recherche Biomédicale, République Démocratique du Congo (RDC); 5 Neurology Department, Michigan State University, East-Lansing, MI, USA; 6 Department of Neurology, Médecine Tropicale, UNIKIN &Institut National de Recherche Biomédicale, République Démocratique du Congo (RDC) and Oregon Institute of Occupational Health Sciences, Oregon Health & Science University, Portland, OR, USA

**Keywords:** Konzo, phénoménologie, malnutrition, aspects socio-économiques, pauvreté, Konzo, phenomenology, malnutrition, socio-economic aspects, poverty

## Abstract

**Introduction:**

Identifier les facteurs déterminant la persistance du konzo à Kahemba en République Démocratique du Congo.

**Méthodes:**

Une enquête transversale a été réalisée à Kahemba en 2011 auprès des ménages de 123 enfants avec konzo (critères OMS) et de 87 enfants sans konzo. La récolte des données s'est faite par interviews, enquête socio-économique par le questionnaire HOME; observation, mesures anthropométriques et examen clinique; mesure du taux de cyanure (CN) dans la farine de manioc et thiocyanate (SCN) urinaire; et analyses sérologiques pour exclure les infections rétrovirales HTLV-I/II et HIV-I/II. L'analyse statistique a été faite par ANOVA, test de Chi-carré, et Kruskall-Wallis au seuil de signification de 0.05.

**Résultats:**

La survenue et la sévérité du konzo étaient associées à la pauvreté des ménages (p < 0,05). Les enfants atteints de konzo présentaient une dégradation nutritionnelle avancée (p < 0,05 ml/l) chez les enfants konzo vs. non-konzo. La population attribuait souvent la maladie à la sorcellerie.

**Conclusion:**

L'intoxication chronique au manioc amer, la malnutrition, ainsi que les croyances superstitieuses favorisent la persistance du konzo à Kahemba. La pauvreté porte le risque d'apparition et de gravité du konzo. Les épidémies de Kahemba dévoilent le risque transgénérationel associé au konzo. L'antécédent de konzo dans la famille élargie constituait un facteur de risque pour la survenue de la maladie (OR= 1,92; p = 0,042). Le taux moyen (±ET) de cyanure dans la farine de manioc était de 92,2 (± 56,2) ppm pour les ménages testés. Les taux moyens (±ET) de SCN urinaire étaient respectivement de 520,4 ± 355,7vs. 382,5± 226,3.

## Introduction

Le konzo est une maladie neurologique chronique dont la survenue a été longtemps attribuée à la consommation de produits alimentaires à base de manioc amer mal détoxifié et à un régime alimentaire pauvre en protéines [1–5]. Sur le plan clinique, la maladie est caractérisée par une paralysie spastique d'installation brutale et irréversible de deux membres inférieurs ou de quatre membres dans les cas sévères [1–5]. La maladie affecte plus les enfants d’âge supérieur à 2 ans et les jeunes femmes en âge de procréer dans plusieurs pays d'Afrique subsaharienne [6, 7]. La maladie survient le plus souvent sous un mode épidémique bien que des cas isolés soient connus. Dans la plupart des cas, la survenue des épidémies a été précédée par des crises socio-économiques perturbant la qualité des vies des populations ainsi que les systèmes existants de sécurité alimentaire. Les cas les plus illustratifs sont ceux des épidémies de Mozambique [8] ainsi que celles de Popokabaka en République Démocratique du Congo(RDC), survenues en périodes de fin de guerre ou d’épidémie de fièvre hémorragique due au virus Ebola [9]. Les études antérieures ont démontré la validité des marqueurs de malnutrition ainsi que ceux d'exposition aux substances cyanhydriques dérivées du manioc dans la prédiction du risque pour le konzo [1–10]. Sur le plan communautaire, la pauvreté et la malnutrition ont été longtemps considérées comme facteur de risque [4]. Malgré la connaissance de tous ces facteurs et l'existence d'un programme national de lutte contre la maladie en RDC, les épidémies de konzo ont continué à survenir, des épidémies actives sont encore signalées même à la rédaction de cette publication. La plus récente épidémie de konzo et la plus grande est celle qui est survenue en 2009 dans la Zone de Santé de Kahemba, Province de Bandundu, RDC [11, 12]. La présente étude avait pour objectif d’élucider le profil épidémiologique du konzo à Kahemba, et d'en déterminer les manifestations particulières et les causes de la persistance de la maladie dans cette contrée.

## Méthodes

Cette étude a consisté en une recherche documentaire et une enquête transversale sur l’épidémie de konzo qui survient de façon répétitive à Kahemba, la zone la plus affectée par le konzo en RDC. Notre approche a été triangulaire pour la collecte des données qui s'est faite par interviews, observations, examen physique des sujets atteints par la maladie, et analyses toxicologiques. Les principales variables d'intérêt étaient (i) la connaissance et le vécu de la maladie par la communauté; (ii) sur le plan sociodémographique le nombre d'enfants par ménage, l’âge et le sexe des enfants ainsi que la présence ou non de la maladie dans la famille restreinte ou élargie; (iii) l'environnement socio-économique familial; et (iv) les manifestations du konzo. L’étude avait reçu l'approbation de Comités d’éthique du Ministère de la santé de la République Démocratique du Congo et de “Oregon Heath and Sciences University ”, Portland, Oregon, Etats Unis d'Amérique.

### Aire d'investigation

**Figure 1:**
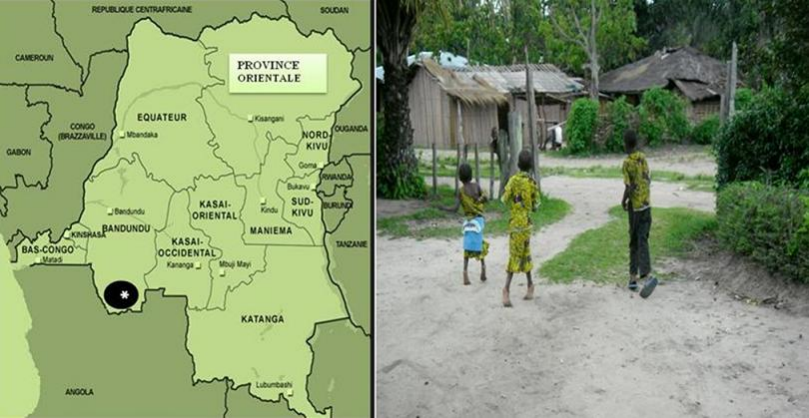
Carte de la République Démocratique du Congo montrant la Zone de Santé de Kahemba en (*) dans la Province de Bandundu au Sud-Ouest du pays à la frontière avec l'Angola, un aspect de l'habitation de la Cité de Kahemba et 3 enfants d'une même fratrie affectés par le konzo

La Cité de Kahemba dans la Zone de Santé de Kahemba, dans la Province de Bandundu au Sud-ouest de la RDC (Figure 1), a été choisie comme site de l’étude du fait de la récurrence et de l'ampleur des épidémies de konzo [11, 12]. Au regard des données actuelles de la littérature [4, 7, 8], ce site est la contrée plus affectée par le konzo dans le monde ces dernières années. Le manioc surtout amer est la principale source de denrées alimentaires pour la grande majorité de la population (∼88,3%) [11]. La Zone de Santé de Kahemba dessert une population d'environ 222.000 habitants et connait un taux de malnutrition aigue de 10% chez les enfants de 0 à 5ans. La prévalence du konzo y a été estimée à 2% dans l'ensemble et jusqu’à 5% dans la Cité de Kahemba [12].

### Participants

Notre enquête a ciblé les ménages de la Cité de Kahemba, le personnel de Santé et les relais communautaires, ainsi que les enfants affectés par le konzo. Ces derniers ont été considérés atteints par le konzo sur base des critères de définition de la maladie tel qu’établis par l'Organisation Mondiale de la Santé [3]: (i) une paraparésie manifeste spastique symétrique d'installation brutale en une semaine, non progressive, chez une personne en bonne santé, (ii) des réflexes ostéo-tendineux exagérés au niveaux rotulien et achilléen, (iii) sans signes d'une autre affection de la moelle. Trois degrés de sévérité de la maladie sont définis de la manière suivante: (i) forme légère pour le malade capable de marcher sans support (stade 1); (ii) forme modérée pour le malade utilisant un ou deux bâtons pour se déplacer (Stade 2), (iii) forme sévère pour le malade incapable de marcher (Stade 3). Les enfants non-konzo ou témoins avaient été résidents des aires de santé de la cité de Kahemba au même moment que les enfants konzo quand la récente épidémie était survenue. Les enfants avec histoire d'hospitalisation pour encéphalopathie (paludisme cérébral, méningo-encéphalite, états de mal épileptique) ont été exclus de l’étude. Des ménages consentants de123 enfants atteints de konzo de 87 enfants sans konzo ont eu à participer.

### Procédures de collecte des données

**Interviews et observations:** Nous avons procédé à des interviews libres ou avec questionnaire auprès des sages de villages, des professionnels de santé, des relais communautaires, ainsi que des membres de familles des ménages recrutés dans notre étude. Les entretiens avec l'Autorité Administrative, le Médecin Chef et l'Infirmier Superviseur de la Zone de Santé, les infirmiers titulaires de Centres de santé, les relais communautaires, et les sages de villages nous ont permis de connaître l'ampleur, la répartition et l’évolution de la maladie dans la Zone de Santé de Kahemba. Des anamnèses familiales ont été réalisées sur base d'un protocole recherchant l’état matrimonial des parents, le mariage consanguin, la structure de la famille; l’âge, le niveau d'instruction et l'occupation des parents, la notion de naissance désirable, la taille de la fratrie, le nombre de personnes affectées par le konzo dans la famille restreinte et élargie; le sexe, l’âge et le niveau scolaire des enfants konzo et non-konzo. Le profil socio-économique a été évalué par le Home Observation for Measurement of the Environment (HOME) selon une version adaptée à l’âge scolaire et aux conditions locales de notre recherche [13, 14]. Grâce à la facilitation de l'infirmier superviseur de la Zone de Santé, nous avons évalué par entretien et observation 10 domaines de l'environnement socio-économique familial à savoir, la qualité et la quantité de l'alimentation, l’élevage, l'approvisionnement en eau, les conditions d'hygiène, la qualité physique de la maison, la densité d'occupation, l’équipement matériel de la maison, le niveau de scolarité de parents, la scolarisation des enfants et la bibliothèque familiale. Les domaines socioéconomiques étaient évalués par le HOME avec des questions dont les réponses étaient cotées soit 0 = inexistant; 1= pauvre; 2= acceptable; 3 = bon. La somme des côtes partielles correspondait à la cote des domaines partiels du HOME tandis que la somme des cotes des 10 domaines correspondait à la cote totale de l'environnement socio-économique familial (Home Total). Le score total score du HOME constituait l'indice socio-économique de la famille.

**Examen physique:** Tous les enfants recrutés dans l’étude ont subi un examen neurologique et somatique approprié. L'examen neurologique a consisté à définir les syndromes moteurs et cognitifs associés au konzo. L'examen somatique a consisté principalement à déterminer l’état nutritionnel des enfants par la mesure des paramètres anthropométriques dont la taille, le poids, le périmètre crânien et le périmètre brachial. Pour apprécier l’état nutritionnel des enfants, les indices Poids/Age, Taille/Age, Poids/Taille, Poids/Age et l'indice de masse corporelle ont été calculés selon les standards de l'OMS ou de NCHS (National Center of Health Statistics) [15].

**Analyses toxicologiques et sérologiques:** Les échantillons de farine de manioc prête à être utilisée pour la préparation du met principal connu sous l'appellation “fufu” ont été collectés auprès de 18 familles consentantes des enfants konzo et non-konzo. Les premières urines du matin ont été recueillies pour chaque enfant. Tous les échantillons ont été congelés dans un thermos à azote liquide et acheminés pour stockage à -80°C avant les analyses. Le thiocyanate urinaire et le cyanure de la farine de manioc ont été mesurés par la méthode semi-quantitative de Howard Bradbury [16, 17] et le résultat était exprimé en micromoles par ml pour le thiocyanate urinaire et en mg par kilogramme de poids pour le cyanure de la farine de manioc. Tous les enfants testés étaient négatifs au HIV-I/II et au HTLV-I/II, cela par le Kit Vironostika (HIV Ag Ag/Ab, Lot A61LY) et le Kit Serodia (Lot: SG 10804).

### Analyses statistiques

Après une analyse descriptive avec calcul des effectifs et des tendances centrales, et de proportions, les tests de χ^2^, l'analyse des variances et les tests non paramétriques de Mann-Whitney et de Kruskal-Wallis ont été appliqués au seuil de signification de p ≤ 0,05. Ces données ont été traitées au moyen du logiciel SPSS (version 17.0).

## Résultats

### Phénoménologie du konzo à Kahemba

#### Connaissance de la maladie par les relais communautaires et la population

D'après les relais communautaires, le konzo affecte plus les populations autochtones de la Cité de Kahemba. Il s'agit de peuples Tchokwe, Lunda et Sonde, qui pratiquent encore le mariage consanguin. Ces populations résisteraient plus à l'action de sensibilisation sur l'origine toxico-nutritionnelle de la maladie et l'attribueraient plus à la sorcellerie. Certains chefs de villages sont accusés de sorciers. Notre entretien avec l'un d'entre eux avait révélé qu'il était aussi père de deux enfants atteints de konzo. Les femmes après accouchement et les enfants sont connus comme les principales victimes de la maladie. Deux cas des enfants ayant connu la maladie avant l’âge de trois ans ont été rapportés. Un des 2 enfants avait été atteint juste après l'acquisition de la marche à l’âge de 8mois et un autre à l’âge de 31mois. La maladie est connue comme une maladie de la saison sèche, plus fréquente entre les mois de juin et de septembre. Le déplacement, pendant cette période de certaines familles au village pour échapper à la maladie, a été rapporté. La population recoure au traitement traditionnel utilisant toutes sortes de plantes pour la rééducation physique et la préparation des potions buvables et, à des mesures diététiques comprenant une alimentation riche en protéines. Paradoxalement, nous avons noté des interdits alimentaires restreignant les possibilités d'apport en protéines. Certains praticiens interdisent la consommation d'oeufs, de grillons, de sauterelles et/ou de champignons. L'efficacité du traitement traditionnel était vantée par la population. Deux femmes adultes et un garçon, ayant connu la maladie au stade III et jugés guéris par le traitement traditionnel, nous ont été présentés. Notre examen neurologique avait retenu chez ces trois personnes une exagération des réflexes ostéo-tendineux. Le changement d'humeur et de caractère, des accès de colère avec agressivité démesurée, la désobéissance, des propos désobligeants et l'insolence ainsi que la jovialité ont été mentionnés comme changement de comportement observé chez les enfants atteints de konzo.

#### Faits d'observation directe

Les enfants konzo apparaissaient ralentis dans la relation duelle. Leurs voix étaient souvent nasonnées. Ils paraissaient petits par rapport à leurs âges. Ils ne marchaient pas tous de la même façon. Trois types de marche ont été observés: (i) marche sur la pointe de pieds sur les deux membres inférieurs, (ii) marche sur la pointe de pieds plus appuyée sur un membre inférieur, et (iii) marche sur la plante de pieds avec un ou deux genoux semi-fléchis. Les enfants konzo se présentaient souvent dans une tenue vestimentaire et physique négligée et sentaient l'odeur de fumée, probablement du fait que le bois est utilisé pour le chauffage des nuits froides. Lors de notre enquête, l'intégration des enfants konzo a été facile au sein de l’équipe de travail, ces derniers étaient prêts à assister les investigateurs notamment dans les préparatifs du matériel de collection d’échantillons. La relation duelle était aussi prompte avec les investigateurs. Un des enfants a affirmé ne pas aller à l’école car son stylo lui été tout le temps «volé» à chaque moment qu'il devait écrire. Lui demandant d'expliquer le phénomène, l'enfant a démontré l'incapacité de tenir son stylo qui se dérobait des ses doigts à chaque tentative de rédaction. Le manioc constituait l'aliment de base. Visiblement, la communauté n'est pas inondée de denrées alimentaires. Cependant, sur le marché local, l'on pouvait observer une variété de légumes par ci par là, on pouvait remarquer des marchands ambulants de fruits (bananes et avocats), rarement de pommes de terre ou de chenilles.

#### Connaissance de la maladie par les professionnels de santé

Les professionnels de santé interrogés connaissaient bien la maladie ainsi que le critérium de l'OMS. Ils avaient mis en place un dispositif de notification et d'enregistrement des cas de konzo. Le nombre de cas recensés de 2004 à 2009 étaient de 3093 pour l'ensemble de la Zone de Santé de Kahemba dont 2046 pour l'an 2009, et 2386 pour la seule Cité de Kahemba[12]. La fréquence la plus élevée des cas enregistrés a été obtenue en 2009 (Tableau 1), cette haute fréquence a été expliquée par le conflit frontalier dans cette zone située entre la RDC et la République d'Angola. Ce conflit avait perturbé en ce moment- là les échanges commerciaux et l'approvisionnement de la Cité Kahemba en produits alimentaires en provenance de la République angolaise. En 2011, la baisse de la fréquence était due entre autres à un travail d'information et de sensibilisation réalisé par l'ONG International Action contre la faim sur la toxicité du manioc insuffisamment roui et séché [11]. Mis à part les critères OMS de dépistage et les aspects nutritionnels, les professionnels de santé de la Cité de Kahemba ont déclaré n'avoir qu'une connaissance limitée sur le konzo. Les malades konzo ne fréquentent pas les structures de soins de santé primaires au début de la maladie ni après, sauf en cas de problèmes de santé en comorbidité avec le konzo. Les structures de soins de santé primaires ne disposent pas de protocole de prise en charge de konzo. Dans l'ensemble de cas recensés dans le système des soins de santé primaires, le sexe féminin représentait 56,9% et les sujets d’âge inférieur à 15ans 79,47%.

**Tableau 1:** Répartition des cas de konzo recensés dans la Zone de Santé de Kahemba, suivant l'année, la tranche d’âge d'apparition de la maladie et le genre, selon le modèle de la Zone de Santé

				Nombre de cas par tranche d’âge et selon le genre								
Année	Nombre Total de cas selon le genre			≤ 5ans			6-14 ans			≥15 ans		
	T	M	F	T	M	F	T	M	F	T	M	F
2004	117	47	70	26	12	14	62	26	36	29	9	20
2005	145	64	81	30	14	16	93	36	57	22	14	8
2006	358	159	199	101	44	57	168	75	93	89	40	49
2007	305	112	193	95	39	56	141	50	91	69	23	46
2008	122	52	70	23	8	15	71	33	38	28	11	17
2009	2046	897	1149	564	244	320	1084	461	623	398	192	206
2010	339	134	205	70	35	35	158	82	76	111	17	94
2011	75	25	50	6	2	4	34	19	15	35	4	31
Total	3507	1490	2017	915	398	517	1811	782	1029	781	310	471

#### Facteurs sociodémographiques et économiques de risque de konzo

***Caractéristiques sociodémographiques et cliniques des enfants konzo et non-konzo:*** Les enfants de parents en situation de mariage représentaient 60,03% des cas et de mariage consanguin 20,6% sans différence entre les konzo et les non-konzo (p > 0,05). Le père a été retrouvé comme chef de ménage dans 66, 8% des cas, la mère dans 14% et les grands-parents dans 13,5%. Le chef de ménage avait surtout comme occupation agriculture dans 40% et enseignement dans 15,9%. Il n'y avait pas de différence statistiquement significative entre les ménages des enfants konzo et non-konzo concernant le nombre d'enfants par ménage (étendue de1 à 13 et moyenne de 6,14) (Tableau 2). Quant à l’âge et genre, l'absence de différence (Tableau 2) était due à la tentative d'appariement au recrutement. L'antécédent de konzo dans la famille restreinte ou élargie était plus fréquent dans les cas des enfants konzo que chez les non-konzo:79,7% contre 67,8% (p = 0,042; OR = 1,92(IC 95%: 1,019-3,587). Selon les critères de degré de sévérité de konzo selon l'OMS, 74.8% d'enfants étaient au stade1, 10.6% au stade 2, et 14, 6% au stade3.

**Table 2:** Caractéristiques sociodémographiques des enfants konzo et non-konzo (page 9)

Caractéristiques	konzo (n = 123)	non-konzo(n = 87)	p
Age en années, moyenne (ET)	8,7(2,5)	9,1(2,6)	.216
Genre Féminin, N (%)	58 (47,2)	65(39,5)	
Sex ratio G:F	1.12:1	1,5: 1	.320
Nombre d'enfants/ménage, moyenne (ET)	6.2 (2.4)	6,1(2,3)	.399
Antécédent de konzo en famille, N (%)	98(79.7)	59 (67,8)	.042

**Figure 2:**
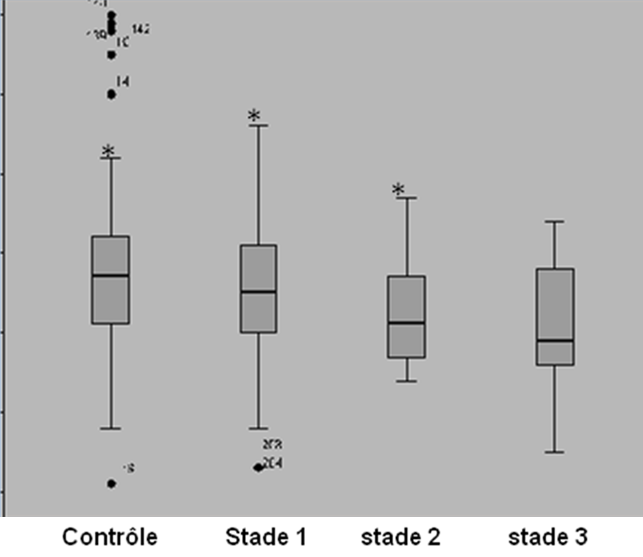
Faibles scores de l'environnement socio-économique familial en lien avec la sévérité du konzo /Critères de l'OMS, scores diminuant respectivement de non-konzo vers le konzo au stade 3. De gauche à droite, du contrôle vers le stade 3 sans, il y avait une différence significative de scores du HOME pour chacun de 3 premiers états (*) Vs stade 3 mais pas entre eux

***Environnement socio-économique familial et état nutritionnel des enfants konzo et non-konzo:*** Il n'y avait pas de différence significative entre les cas konzo et les non-konzo dans 6 domaines de l'environnement socio-économique familial à savoir: la possession des biens matériels; l'approvisionnement eau; l’élevage, la qualité de la maison, l'hygiène de l'habitat et la bibliothèque familiale. Par contre, des différences étaient observées en défaveur des enfants konzo dans les 4 autres domaines restants notamment la quantité et la qualité de la nourriture, la densité d'occupation de la maison, la scolarité des enfants et le niveau d'instruction des parents (p < 0,05)(Tableau 3). Le Tableau 3 montre aussi des états nutritionnels dégradés des enfants konzo par rapport aux non-konzo dans toutes les modalités évaluatives (p ≤ 0,001) sauf pour l'indice de masse corporelle (p= 0,189). La sévérité du konzo était associée aux cotes basses de l'environnement socio-économique familial global (p = 0,008; Kruskall-Wallis) (Figure 2).

**Tableau 3:** Environnement socio-économique familial (HOME) et état nutritionnel des enfants konzo et non-konzo

Domaines du HOME	Etat neurologique des enfants		
	Konzo	non-konzo	p ( Test Mann-Whitney
Home total, Médiane (IIQ)	44(12)	47(11)	.009
Qualité et quantité nourriture, Médiane(IIQ)	8(2)	9(3)	.042
Densité Occupation de la maison,Médiane (IIQ)	3(1)	3(2)	.008
Scolarité des enfants,Médiane (IIQ)	3(1)	3(0)	.001
Scolarité des parents, Médiane(IIQ)	8(4)	8.5(4)	.014
Indices nutritionals des enfants	Konzo	Non-konzo	P
WAZ-NCHS, Moyenne (ET)	-2.850187 ( 0.9758525)	**-**2.133047 (1.4975538)	<0,001
HAZ-WHO, Moyenne (ET)	-3,356707 (1,3531059)	-2,326224 (1,8036608)	<0,001
HAM-NCHS, Moyenne (ET)	85,067480 (6,0890976)	89,510588 (8,0106018)	<0,001
HEIGHT-AGE-WHO, Moyenne (ET)	63,878211 (23,5914385)	78,549770 (26,4734532)	< 0,001
BMIZ-WHO, Moyenne (ET)	-1,975325 (1,5835197)	-1,670353 (1,6767690)	0,189

**II Q** = Intervalle interquartile; **ET** = Ecart-type; **WAZ**=Indice Poids-pour- l’âge Z-score; **HAZ**=Indice Taille-pour- l’âge Z score; **BMIZ**=Indice de masse corporelle Z-score; Height-Age = Indice Taille-pour-âge; **NCHS**= National Center of Health Statistics, USA.

#### Résultats des analyses toxicologiques

Tant pour les cas konzo que pour les non-konzo, la valeur moyenne de thiocyanate urinaire était supérieure au seuil limite d'absence de risque de konzo de 100 micromoles par litre (OMS), soit 382,48± 226,30 micromoles par litre pour les non-konzo et 520,43±355,66 micromoles par litre pour les konzo [11]. Le taux de cyanure dans les échantillons de la farine de manioc consommée variait entre 30 et 200mg de HCN/kg. Le taux était de 50 à 200mg/kg pour 16 échantillons sur 18 (88,9%), de 100 à 200mg pour 10 (55,6%). La moyenne (±écart-type) du taux de cyanure dans la farine de manioc était de 92,22 (±56,21) mg de HCN/kg de poids. Ces taux indiquaient bien une exposition à l'intoxication cyanhydrique au regard de la norme de sécurité de FAO/OMS de 10 mg par kg de poids sec [18].

## Discussion

### Facteurs déterminant le konzo et sa persistance à Kahemba

Les facteurs associés au konzo et ses épidémies à Kahemba sont d'emblée comparables à ceux mentionnés dans la littérature, à savoir la saison sèche et l'insécurité alimentaire associée aux conflits armés ou à d'autres formes d'insécurité sociale. Les épidémies de Popokabaka et de Bukavu en RDC [2, 19], celles de Mozambique et du Cameroun en sont illustratives [8, 20]. La saison sèche correspond à la période où la quantité de la linamarine, substance cyanogénétique, est très élevée dans le manioc [21], l'insécurité alimentaire favorise la malnutrition aigue qui est un facteur déclenchant du konzo car aggravant le déficit en protéines et favorisant avec l'insécurité sociale la consommation du manioc amer insuffisamment mal détoxifié [11]. La quantité élevée de cyanure trouvée dans quelques échantillons de farine de manioc consommée à Kahemba ainsi que celle de thiocyanate urinaire montraient les risques d'intoxication cyanhydrique auxquels sa population était exposée [11, 18, 22]. Par rapport à tous ces éléments, la situation de Kahemba est comparable à celle du Mozambique [8]. Mais à Kahemba, les populations autochtones les plus affectées pratiquent encore le mariage consanguin et n'attribuent pas la maladie à l'intoxication cyanhydrique mais à la sorcellerie, la mauvaise qualité de l'eau et à l'altération du manioc par le sol [23]. Ils résistent à l'action de sensibilisation sur l'origine toxico-nutritionnelle du konzo. Cette causalité culturelle locale du konzo peut être considérée comme un risque biopsychosocial associé à la persistance du konzo à Kahemba. Ce risque mérite aussi une attention pour ces populations pratiquant la consanguinité dont la preuve biologique reste encore à déterminer d'autant plus que la maladie est observée en grappes au sein de communautés familiales.

### Impact de l'environnement socio-économique du konzo

**Figure 3:**
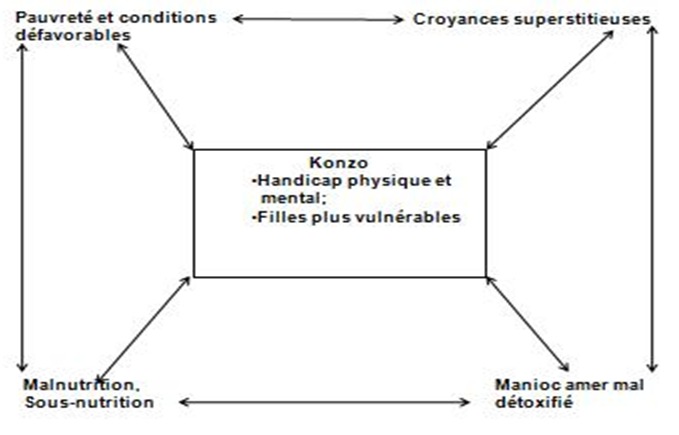
Modèle circulaire de vulnérabilité socio-économique au konzo: la pauvreté, les conditions défavorables, les croyances superstitieuses, la consommation du manioc amer mal détoxifié et la sous-nutrition constituent l’écologie du konzo, un handicap physique et mental affectant plus les filles. Les facteurs déterminants de la maladie portent un risque transgénérationnel qui mérite des études plus approfondies

Les données de notre étude ont démontré, comme dans la littérature [1–11, 19–21], que le konzo était due à l'exposition à l'intoxication cyanhydrique chronique modérée ou sévère, la malnutrition chronique et la pauvreté. La pauvreté, caractérisée l'insuffisance de nourriture, la promiscuité et le niveau d'instruction bas, s'est avérée comme un autre risque favorisant le konzo à Kahemba. La sévérité du konzo était en lien avec le niveau élevé de détresse socio-économique familiale. Ainsi, dans l'intoxication cyanhydrique diététique, le niveau élevé de détresse socio-économique des familles porte un double risque, de la survenue du konzo et de sa gravité. Les aspects phénoménologiques et socio-économiques du konzo tels décrits dans notre étude montrent que le konzo à Kahemba engage dans son origine et ses manifestations des facteurs socio-économiques, psychosociaux (pauvreté, insécurité sociale, handicap chronique, croyances superstitieuses) et toxico-nutritionnels (malnutrition, faim et intoxication cyanhydrique). Ce qui expliquerait son polymorphisme et sa complexité. En considérant les résultats d'une récente publication sur les troubles neuropsychologiques dans le konzo, affectant plus gravement les filles [24], ces aspects phénoménologiques et socio-économiques du konzo laissent suggérer un modèle circulaire de vulnérabilité socio-économique et transgénérationnelle au konzo (Figure 3). Ce modèle peut guider de nouveau la recherche, la prévention et le traitement du konzo. En outre, les populations de Kahemba assurent une bonne insertion communautaire des patients et utilisent toutes sortes de moyens traditionnels dont des mesures diététiques pour prendre en charge les patients. Elles croient à la réversibilité de déficiences dues au konzo. Cette référence culturelle peut servir pour la recherche-action, les études anthropologiques et l'intervention psychosociale dans la prévention du konzo.

## Conclusion

L'intoxication cyanhydrique chronique par le manioc amer mal détoxifié, la pauvreté, la malnutrition, les croyances superstitieuses soutiennent la persistance du konzo à Kahemba. La détresse socio-économique porte le risque d'apparition et de gravité du konzo.
